# The effect of iterative reconstruction on computed tomography assessment of emphysema, air trapping and airway dimensions

**DOI:** 10.1007/s00330-012-2489-z

**Published:** 2012-05-23

**Authors:** Onno M. Mets, Martin J. Willemink, Freek P. L. de Kort, Christian P. Mol, Tim Leiner, Matthijs Oudkerk, Mathias Prokop, Pim A. de Jong

**Affiliations:** 1 Department of Radiology, UMC Utrecht, P.O. Box 85500, E01.132, 3508 GA Utrecht, the Netherlands; 2 Utrecht University Medical Center, Image Sciences Institute, Utrecht, the Netherlands; 3 Department of Radiology, Groningen University Medical Center, Groningen, the Netherlands; 4 Department of Radiology, Nijmegen University Medical Center, Nijmegen, the Netherlands

**Keywords:** Computed tomography, Iterative reconstruction, Pulmonary emphysema, Chronic obstructive pulmonary disease, Quantitative CT

## Abstract

**Objectives:**

To determine the influence of iterative reconstruction (IR) on quantitative computed tomography (CT) measurements of emphysema, air trapping, and airway wall and lumen dimensions, compared to filtered back-projection (FBP).

**Methods:**

Inspiratory and expiratory chest CTs of 75 patients (37 male, 38 female; mean age 64.0 ± 5.7 years) were reconstructed using FBP and IR. CT emphysema, CT air trapping and airway dimensions of a segmental bronchus were quantified using several commonly used quantification methods. The two algorithms were compared using the concordance correlation coefficient (*p*_c_) and Wilcoxon signed rank test.

**Results:**

Only the E/I-ratio_MLD_ as a measure of CT air trapping and airway dimensions showed no significant differences between the algorithms, whereas all CT emphysema and the other CT air trapping measures were significantly different at IR when compared to FBP (*P* < 0.001).

**Conclusion:**

The evaluated IR algorithm significantly influences quantitative CT measures in the assessment of emphysema and air trapping. However, the E/I-ratio_MLD_ as a measure of CT air trapping, as well as the airway measurements, is unaffected by this reconstruction method. Quantitative CT of the lungs should be performed with careful attention to the CT protocol, especially when iterative reconstruction is introduced.

***Key Points*:**

• *New techniques in CT allow numerous quantitative measurements of lung function*.

• *Iterative reconstruction influences quantitative CT measurements of emphysema and air trapping*.

• *Expiratory-to-inspiratory ratio of mean lung density and airway measurements are unaffected by iterative reconstruction*.

• *Quantitative lung-CT should be performed with careful attention to the CT protocol*.

## Introduction

The use of computed tomography (CT) increases rapidly, resulting in a marked increase in radiation exposure for the population [1]. Therefore, radiation dose saving has received much attention and has been pursued by introducing low-dose protocols using the conventional filtered back-projection (FBP) algorithm for image reconstruction. However, the constraint on radiation dose increases image noise [2]. Recent advances in computational power allowed the introduction of iterative reconstruction (IR) algorithms for image reconstruction. Data suggest that IR allows radiation dose reduction by 50 % or more compared to standard dose acquisition, while maintaining image quality [3–6]. Such a dose reduction would be a major step forward, especially in case of repeated evaluations and follow-up as regularly applied in chest imaging. However, the influence of IR on quantitative CT measurements, e.g. measurement of lung density and airway dimensions used in the evaluation of chronic obstructive pulmonary disease (COPD), is not yet known. Therefore, the aim of this study was to determine the influence of IR on quantitative CT measurements of pulmonary emphysema, air trapping and airway dimensions, compared to the standard FBP algorithm.

## Materials and methods

### Subjects

This study was performed in subjects participating in the population-based Dutch and Belgian randomized lung cancer screening trial (NELSON trial). Inclusion criteria of the trial and study population characteristics have previously been described in detail [7]. Briefly, participants were at baseline current and former smokers (who quit no more than 10 years ago) between the age of 50 and 75 years, who smoked more than 15 cigarettes per day during more than 25 years or more than 10 cigarettes per day during more than 30 years. In the present study we included 83 consecutive subjects who received a paired inspiratory and expiratory CT between June 2011 and August 2011 for lung cancer screening purposes. All CTs were reconstructed using both standard FBP and IR. We excluded a total of eight subjects owing to CT protocol violation (*n* = 1), post-operative changes after lobectomy of the right upper lobe (*n* = 1), and failure of the automatic lung segmentation (*n* = 6) (see Sect. “Quantitative analysis of emphysema and air trapping”). The final study population thus comprised 75 subjects.

### CT data acquisition and image reconstruction

Chest CT was performed using one of two available CT systems: 44 subjects were examined using 64-slice CT (Brilliance 64; Philips Healthcare, Best, the Netherlands) with a smooth reconstruction filter (C-filter, Philips); 31 subjects were examined using 256-slice CT (Brilliance iCT; Philips Healthcare, Best, the Netherlands) using either a smooth B-filter (*n* = 24) or C-filter (*n* = 7). Slices of 1 mm thickness with 0.7-mm increment were reconstructed. Dose settings were adjusted to patients body weight: 120 kVp at 30 mAs for inspiratory CT and 80 kVp at 30 mAs for expiratory CT in subjects weighing less than 80 kg, and 140 kVp at 30 mAs for inspiratory CT and 120 kVp at 20 mAs for expiratory CT in subjects weighing 80 kg or more.

Raw CT data of the study subjects were reconstructed using both standard FBP and hybrid IR (iDose; Philips Healthcare, Best, the Netherlands). iDose is a recently introduced reconstruction algorithm using two denoising components [3, 6, 8], which provides image noise reduction without changing the image characteristics. Technically, iDose applies an iterative maximum likelihood denoising algorithm, based on Poisson statistics, on the raw projection data. Subsequently, the reconstructed images are iteratively adjusted in order to decrease uncorrelated noise. The level of noise reduction is adjustable by selecting one of seven levels (with level 1 having the least noise reduction, and level 7 having the most noise reduction). iDose level 6 was used in the present study, resulting in a theoretical noise reduction of 45 % compared to FBP [8].

### Quantitative analysis of emphysema and air trapping

Specialized software automatically segmented the lungs from the chest wall, mediastinum, diaphragm and airways [9] in the inspiratory and expiratory CT images of both reconstruction algorithms. Additionally, all lung segmentation results were visually checked and those with major errors excluded, as previously described [10]. Attenuation of each voxel within the segmented lung volume was assessed, and several commonly used CT emphysema and CT air trapping measures were calculated from the attenuation distribution histogram. CT emphysema was defined as the percentage of voxels below −950 HU in inspiratory CT (IN_−950_) [11] and as the Hounsfield unit (HU) value at the 15th percentile of the attenuation curve (Perc_15_) [12]. CT air trapping was defined as the percentage of voxels below −856 HU in expiratory CT (EXP_−856_) [13], the change in relative lung volume with attenuation values between −860 and −950 HU (RVC_−860 to −950_) [14], and as the expiratory to inspiratory ratio of the mean lung density (E/I-ratio_MLD_) [15].

### Quantitative analysis of airway dimensions

We used the apical segmental bronchus of the right upper lobe (RB1) to assess airway dimensions [16]. Airway dimensions of RB1 were measured using validated custom software based on the full-width-at-half maximum method (EmphylxJ; University of British Columbia, Vancouver, BC, Canada) [16, 17]. In short, the RB1 was visually identified on the inspiratory CT by a trained observer, who manually placed a seed point in the lumen centre. The software then calculates the x-ray attenuation along rays placed from the lumen centre outwards in all directions. The airway boundaries are assumed halfway to the maximum on the lumen side, and halfway to the minimum on the parenchymal side [17, 18]. Using these airway wall boundaries we calculated absolute values of lumen area (LA), wall area (WA) and internal perimeter (Pi) of the RB1 for each subject. Additionally, wall area was expressed as percentage of total airway area: 100 % × WA/(WA + LA) = WA%. The airway measurements were performed similarly and at the exact same location in both inspiratory CT series.

### Data analysis

Quantitative CT measures of emphysema, air trapping and airway dimensions for the conventional FBP and the IR algorithm were compared within each subject. The agreement of the quantitative CT measures using the two algorithms was assessed by the concordance correlation coefficient (*p*_c_), which takes into account both the correlation and the distance to the line of identity [19]. A *p*_c_ value less than 0.90 was considered to represent poor agreement, whereas higher *p*_c_ values represent moderate (0.90 ≤ *p*_c_ ≤ 0.95), substantial (0.95 < *p*_c_ ≤ 0.99) or almost perfect (*p*_c_ > 0.99) agreement, based on the descriptive scale for continuous variables [20]. The non-parametric Wilcoxon signed rank test was used in all variables to test for statistical differences within the subjects.

Statistical analyses were performed using SPSS software v15.0 (SPSS Inc, Chicago, Illinois, USA) and MedCalc v11.3.8.0, Mariakerke Belgium. A *P* value below 0.05 was considered statistically significant. Values given are medians with interquartile range, unless indicated otherwise.

## Results

Subjects in our study population were on average 64.0 ± 5.7 (SD) years of age, and male (*n* = 37, 49 %) and female subjects (*n* = 38, 51 %) were equally represented.

### Comparison of quantitative CT measures

**Fig. 1:**
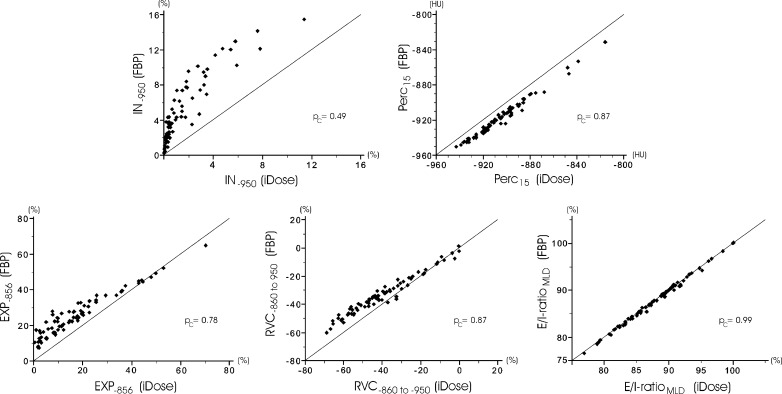
Scatter plots of quantitative CT measures of emphysema and CT air trapping using conventional filtered back-projection (FBP) and hybrid iterative reconstruction (iDose). Structural differences between iDose and FBP are shown for CT emphysema as percentage of voxels below −950 HU (IN_−950_) and as 15th percentile of attenuation distribution curve (Perc_15_) (*upper row*) and CT air trapping as percentage of voxels below −856 HU (EXP_−856_) and as relative change in lung volume with attenuation between −860 and −950 HU (RVC_−860 to −950_) (*lower left and middle*). The only quantitative CT measure with a concordance correlation coefficient (*p*_c_) of at least 0.90, and thus insensitivity to the iterative reconstruction, is the expiratory to inspiratory ratio of mean lung density (E/I-ratio_MLD_, *lower right*)

**Fig. 2:**
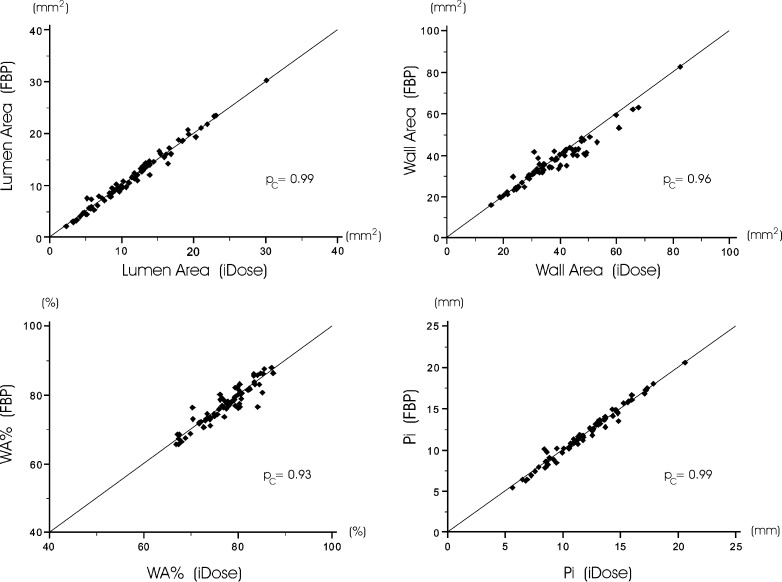
Scatter plots of quantitative CT measures of the apical segmental bronchus of the right upper lobe using conventional filtered back-projection (FBP) and hybrid iterative reconstruction (iDose). No structural differences between iDose and FBP were found for lumen area (*upper left*), wall area (*upper right*), wall area percentage (WA%, *lower left*) and internal perimeter (Pi, *lower right*), because all concordance correlation coefficients (*p*_c_) were at least 0.90

**Fig. 3:**
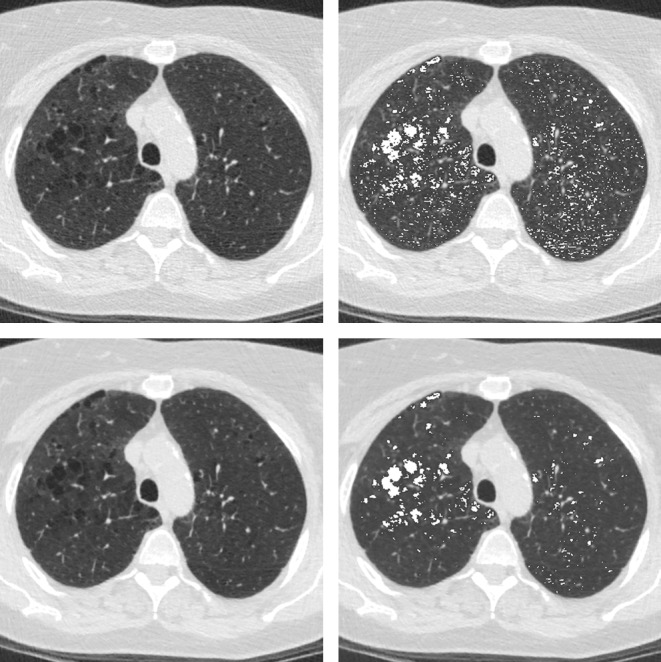
Quantitative assessment of CT emphysema using the conventional filtered back-projection (FBP) and iterative reconstruction algorithm (iDose). Axial CT images in inspiration. The lungs are automatically segmented from the chest wall, airways and mediastinum using dedicated software. Attenuation of each voxel in the segmented lung volume is calculated and CT emphysema is defined as voxels with an attenuation below −950 HU; voxels within this range are coloured *white* (*right images*). Note the denoising effect and the difference in CT emphysema between the FBP (*upper images*) and iDose (*lower images*)

Comparison of quantitative CT measures using FBP and IR showed significant differences for all CT emphysema measurements. Also most CT air trapping measures differed significantly between FBP and IR, except for the E/I-ratio_MLD_ as a measure of CT air trapping. Finally, airway measurements showed no significant differences between the algorithms. Table 1 lists the quantitative results per reconstruction algorithm. The absolute differences in CT emphysema were 3.04 % (interquartile range 1.86–4.62) for IN_−950_ and 11 HU (interquartile range 10–13) for Perc_15_. The absolute differences in CT air trapping were 8.0 % (interquartile range 6.1–11.2) for EXP_−856_ and 7.6 % (interquartile range 4.2–10.2) for RVC_−860 to −950_. Except for E/I-ratio_MLD_, all CT measures of emphysema and air trapping showed poor agreement between standard FBP and IR. CT measures of the apical segmental bronchus of the right upper lobe showed on average substantial agreement. The results of quantitative CT assessment using both reconstruction algorithms are presented in Figs. 1 and 2, further illustrating the systematic differences. Figure 3 shows an example of quantitative assessment of CT emphysema using either the conventional FBP and the IR algorithm.

**Table 1:** Differences in quantitative CT measurements of emphysema, air trapping and airway dimensions using filtered back-projection (FBP) and iDose reconstruction algorithms

	FBP	iDose	*p*_c_ value	*P* value
CT emphysema				
IN_−950_ (%)	3.81 (2.17–7.46)	0.57 (0.25–2.26)	0.486	<0.001
Perc_15_ (HU)	−918 (−907 to −931)	−906 (−896 to −920)	0.866	<0.001
CT air trapping				
EXP_−856_ (%)	24.3 (17.0–32.6)	14.4 (7.4–22.3)	0.777	<0.001
RVC_−860 to −950_ (%)	−35.3 (−43.1 to −26.3)	−42.6 (−54.7 to −30.6)	0.873	<0.001
E/I-ratio_MLD_ (%)	87.9 (83.9–90.9)	88.1 (84.3–90.8)	0.998	NS
Airway measurements				
Lumen area (mm^2^)	10.3 (7.6–14.6)	10.2 (7.3–14.5)	0.991	NS
Wall area (mm^2^)	34.9 (29.4–41.7)	34.4 (28.8–44.3)	0.960	NS
WA% (%)	77.0 (73.1–81.4)	77.6 (73.4–80.7)	0.935	NS
Pi (mm)	11.8 (10.2–13.8)	11.7 (10.0–13.7)	0.990	NS

Values given are median with interquartile range

*HU* Hounsfield units; *IN*_*−950*_ CT emphysema as percentage of voxels below −950 HU; *Perc*_*15*_ CT emphysema as 15th percentile of attenuation distribution curve; *EXP*_*−856*_ CT air trapping as percentage of voxels below −856 HU; *RVC*_*−860 to −950*_ CT air trapping as relative change in lung volume with attenuation between −860 and −950 HU; *E/I-ratio*_*MLD*_ expiratory to inspiratory ratio of mean lung density; *WA%* wall area percentage as 100 % × WA/(WA + LA); *Pi* internal perimeter of the airway; *p*_*c*_ *value* concordance correlation coefficient, a correlation <0.90 represents poor agreement (see Figs. 1 and 2 for a visual representation); *NS* not significant

## Discussion

This study found that noise-reducing IR significantly alters most of the quantitative measures of CT emphysema and CT air trapping generally used in respiratory research. However, it seems that the E/I-ratio_MLD_ as a measure of CT air trapping and quantitative measurements of a relatively large airway lumen and wall remain unchanged between the two reconstruction algorithms. These findings may be important given that CT quantification of the lungs is increasingly used, while dose reduction and IR methods are introduced at the same time.

Previous studies have shown that quantitative CT emphysema measures are influenced by several technical factors such as slice thickness [21, 22] and type of CT equipment used [23]. However, research into the factors which may influence CT air trapping assessment and airway measurements has been limited [18]. Our study offers insight into the influence of IR in several widely used quantitative CT measures of emphysema, air trapping and airway dimensions. Given the significant differences that we have shown between the two reconstruction methods for several measures, our findings underline that comparison of quantitative CT results of lung densitometry should always be performed with careful attention to the protocols used for CT data acquisition and image reconstruction/analysis [18, 24].

Regarding the application of CT air trapping, our findings suggest that E/I-ratio_MLD_ is the preferred method given its insensitivity to differences in the evaluated reconstruction algorithms. This insensitivity may be due to the fact that denoising in IR affects the extremes of the attenuation distribution histogram. As a consequence, threshold methods (e.g. EXP_−856_ or RVC_−860 to −950_ for CT air trapping) are substantially altered whereas the mean lung density is hardly affected and this in combination with the use of an inspiratory to expiratory ratio apparently makes this measure independent of a denoising algorithm. If this insensitivity also applies to other protocol differences, such as kVp and mAs, this might imply that E/I-ratio_MLD_ is preferable over other CT air trapping measures.

Further, our findings suggest that the denoising process in IR does not affect the delineation of segmental airway structures, given that the differences in airway measurements for the right apical segmental bronchus between both reconstruction methods were not significantly different from zero.

Our study has potential limitations. Firstly, it is important to note the lack of a pathological reference standard, which would be needed to judge which method is close to a ‘pathological truth’, although we would like to emphasize that this study specifically aimed to investigate and describe the differences that occur when IR is applied instead of conventional FBP. Secondly, it is noted that our results might differ between altering IR denoising levels and other IR algorithms and CT manufacturers, given that the results were obtained from testing the IR algorithm of a single vendor at one denoising level. Future research might focus on ways to correct for structural differences in quantitative measures when IR algorithms are applied. Thirdly, we focussed on a commonly used segmental airway and on the basis of our findings it cannot be concluded that measurements on smaller airways are unaffected by IR.

In conclusion, our study shows that the evaluated IR algorithm significantly alters quantitative CT measures in the assessment of all emphysema and most commonly used air trapping measures, compared to FBP. However, both the E/I-ratio_MLD_ as a measure of CT air trapping and the quantitative measurements in a segmental airway are unaffected by this reconstruction method. Quantitative CT lung densitometry should always be performed with careful attention to the CT protocol, especially in an era of increased use of quantitative CT where dose reduction and iterative reconstruction are introduced.

## Abbreviations and acronyms

COPD: chronic obstructive pulmonary disease
CT: computed tomography
E/I-ratio_MLD_: expiratory to inspiratory ratio of the mean lung density
EXP_−856_: percentage of voxels below −856 HU in expiratory CT
FBP: filtered back-projection
HU: Hounsfield unit
IN_−950_: percentage of voxels below −950 HU in inspiratory CT
IR: iterative reconstruction
LA: lumen area
NELSON trial: Nederlands Leuvens Longkanker Screenings Onderzoek
p_c_: concordance correlation coefficient
Perc_15_: 15th percentile of the attenuation curve
Pi: internal perimeter
RB1: right upper lobe
RVC_−860 to −950_: change in relative lung volume with attenuation values between −860 and −950 HU
SD: standard deviation
WA: wall area

## References

[1] Brenner DJ, Hall EJ (2007). Computed tomography–an increasing source of radiation exposure. N Engl J Med.

[2] Yuan R, Mayo JR, Hogg JC (2007). The effects of radiation dose and CT manufacturer on measurements of lung densitometry. Chest.

[3] Funama Y, Taguchi K, Utsunomiya D (2011). Combination of a low-tube-voltage technique with hybrid iterative reconstruction (iDose) algorithm at coronary computed tomographic angiography. J Comput Assist Tomogr.

[4] Winklehner A, Karlo C, Puippe G (2011). Raw data-based iterative reconstruction in body CTA: evaluation of radiation dose saving potential. Eur Radiol.

[5] Sagara Y, Hara AK, Pavlicek W, Silva AC, Paden RG, Wu Q (2010). Abdominal CT: comparison of low-dose CT with adaptive statistical iterative reconstruction and routine-dose CT with filtered back projection in 53 patients. AJR Am J Roentgenol.

[6] Habets J, Symersky P, de Mol BA, Mali WP, Leiner T, Budde RP (2011) A novel iterative reconstruction algorithm allows reduced dose multidetector-row CT imaging of mechanical prosthetic heart valves. Int J Cardiovasc Imaging. doi:10.1007/s10554-011-9954-7 10.1007/s10554-011-9954-7 PMC3463798 22002686

[7] van Iersel CA, de Koning HJ, Draisma G (2007). Risk-based selection from the general population in a screening trial: selection criteria, recruitment and power for the Dutch-Belgian randomised lung cancer multi-slice CT screening trial (NELSON). Int J Cancer.

[8] Noel PB, Fingerle AA, Renger B, Munzel D, Rummeny EJ, Dobritz M (2011). Initial performance characterization of a clinical noise-suppressing reconstruction algorithm for MDCT. AJR Am J Roentgenol.

[9] van Rikxoort EM, de Hoop B, Viergever MA, Prokop M, van Ginneken B (2009). Automatic lung segmentation from thoracic computed tomography scans using a hybrid approach with error detection. Med Phys.

[10] Mets OM, Murphy K, Zanen P (2012). The relationship between lung function impairment and quantitative computed tomography in chronic obstructive pulmonary disease. Eur Radiol.

[11] Gevenois PA, de Maertelaer V, De Vuyst P, Zanen J, Yernault JC (1995). Comparison of computed density and macroscopic morphometry in pulmonary emphysema. Am J Respir Crit Care Med.

[12] Newell JD, Hogg JC, Snider GL (2004). Report of a workshop: quantitative computed tomography scanning in longitudinal studies of emphysema. Eur Respir J.

[13] Regan EA, Hokanson JE, Murphy JR (2010). Genetic epidemiology of COPD (COPDGene) study design. COPD.

[14] Matsuoka S, Kurihara Y, Yagihashi K, Hoshino M, Watanabe N, Nakajima Y (2008). Quantitative assessment of air trapping in chronic obstructive pulmonary disease using inspiratory and expiratory volumetric MDCT. AJR Am J Roentgenol.

[15] O'Donnell RA, Peebles C, Ward JA (2004). Relationship between peripheral airway dysfunction, airway obstruction, and neutrophilic inflammation in COPD. Thorax.

[16] Nakano Y, Muro S, Sakai H (2000). Computed tomographic measurements of airway dimensions and emphysema in smokers. Correlation with lung function. Am J Respir Crit Care Med.

[17] Nakano Y, Whittall KP, Kalloger SE, Coxson H, Pare PD, English JC (2002). Development and validation of human airway analysis algorithm using multidetector row CT. Proc SPIE.

[18] Mets OM, de Jong PA, van Ginneken B, Gietema HA, Lammers JW (2012) Quantitative computed tomography in COPD: possibilities and limitations. Lung 190:133–145 10.1007/s00408-011-9353-9 PMC3310986 22179694

[19] Lin LI (1989). A concordance correlation coefficient to evaluate reproducibility. Biometrics.

[20] McBride GB (2005) A proposal for strength-of-agreement criteria for Lin’s concordance correlation coefficient. NIWA Client Report: HAM2005-062. http://www.medcalc.org/download/pdf/McBride2005.pdf. Accessed 14 May 2012

[21] Madani A, Zanen J, De Maertelaer V, Gevenois PA (2006). Pulmonary emphysema: objective quantification at multi-detector row CT - comparison with macroscopic and microscopic morphometry. Radiology.

[22] Gierada DS, Bierhals AJ, Choong CK (2010). Effects of CT section thickness and reconstruction kernel on emphysema quantification relationship to the magnitude of the CT emphysema index. Acad Radiol.

[23] Bakker ME, Stolk J, Putter H (2005). Variability in densitometric assessment of pulmonary emphysema with computed tomography. Invest Radiol.

[24] Coxson HO, Rogers RM (2005). Quantitative computed tomography of chronic obstructive pulmonary disease. Acad Radiol.

